# Priming of *Citrullus lanatus var.* Colocynthoides seeds in seaweed extract improved seed germination, plant growth and performance under salinity conditions

**DOI:** 10.1038/s41598-023-38711-8

**Published:** 2023-07-23

**Authors:** Asmaa M. Radwan, Entesar A. Ahmed, Abdelraheim M. Donia, Abeer E. Mustafa, Mohamed A. Balah

**Affiliations:** 1 Botany and Microbiology Department, Faculty of Science, Girls Branch, Al-Azhar University, Cairo, Egypt; 2 Medicinal and Aromatic Plants Department, Desert Research Center, Cairo, Egypt; 3 Plants Protection Department, Desert Research Center, Cairo, Egypt

**Keywords:** Physiology, Plant sciences

## Abstract

*Citrullus lanatus* var. Colocynthoide “Gurum” is an unconventional crop that can be utilized as a new source of edible oil and has the ability to grow in a variety of harsh conditions. To mitigate the adverse effects of salinity on seed germination and plant performance of *C. lanatus*, seeds were primed in the aqueous extracts of the seaweed *Ulva lactuca* before planting under greenhouse conditions. The aqueous extract of *U. lactuca* at 8% w/v led to maximal seed germination percentage and seedling growth of *C. lanatus*. Moreover, *U. lactuca* extract counteracted the negative effects of salt stress on the plant by significantly increasing the activity of SOD, CAT, and POD. The bioactive components of *U. lactuca,* e.g*.* glycine betaine and phenolic compounds can account for such beneficial role of algal extract on *C. lanatus*. Thus, priming of *C. lanatus* seeds in *U. lactuca* extract with various concentrations of *U. lactuca* extract can be employed as an effective practice for successful seed germination, improved plant growth and enhanced salt resistance, probably as a result of increased antioxidant enzymes activity and photosynthetic pigments.

## Introduction

Salinity is one of the widely spread agricultural problems all over the world that can severely limit crop production^1,2^. It is estimated that up to 20% of the irrigated lands in worldwide are affected different extents by salinity^3^, while 2.1% of the arid lands are suffering from salt stress, and the problem is aggravating^4^. Salinity stress decreases plant growth by adversely affecting various physiological and biochemical processes^5^. Salinity stress decreases photosynthesis rate with a significant reduction in the chlorophyll content which eventually resulted in reduced sunflower yield^6^. One of the physiological changes occurring when plants are exposed to stress conditions is the enhanced production of reactive oxygen species (ROS)^7^. Excessive salt ions can exert dangerous effects like ion toxicity, osmotic stress, and nutritional imbalance^8,9^, which eventually lead to decreased growth and yield^10^. To cope with salinity stress, plants experience increased activity of antioxidant enzymes^11^.

Seed germination is a critical developmental phase in the life cycle of plants^12^ and is a major limiting factor for establishing plants under salinity conditions^13^. Salt stress affects percentage and rate of germination, as well as the subsequent seedling growth in different ways depending on plant species^14^. The impact of stress arising from more than one salt on seed germination is less severe than that arising from a single salt^15,16^. To overcome the impact of salt stress on plant growth and performance, seed priming can be an efficient and economical technique during the early stage of plant life^17,18^. Seed priming is a process in which seeds are hydrated in different solutions of natural or synthetic compounds^17^. Algal extracts have priming effects that can increase seed germination and plant growth under the impact of salinity and other abiotic stresses^19,20^.

Algae are rich in biologically active components, that have the potential to be used as soil cleansing agents, biofertilizers, plant growth promoters and can help in soil cleansing and; fertilization, and plant protectants agents from biotic and abiotic stress factors^20–22^. Due to their plentiful content of biostimulants, algae have been employed to boost plant performance and resistance to environmental stresses due to their plentiful biostimulant chemicals, plant performance, and tolerance to environmental stresses^23^. Bioactive compounds derived from marine algae have recently been used as biofertilizers for crops to increase the magnitude and yield of agricultural and horticultural crops and quality while minimizing environmental impacts^24^. Seaweed extracts contain a variety of growth hormones, polysaccharides, and macro- and micronutrients^25^. The extract of the seaweed *Ulva rigida* improved salt stress resistance and protect plants from oxidative damage arising from abiotic stress^23,26^.

*Citrullus lanatus* var. Colocynthoides (Gurum), family Cucurbitaceae, locally known as seed watermelon or “Gorma” is a promising fodder and oil crop in Egypt. The foliage of the plant is used as animal feed and its seeds have been investigated as an alternative source of vegetable oil^27^ and as a new source of high-quality pectin for commercial utilization^28^. However, seed germination and plant growth of *C. lanatus* are affected adversely by salinity. Meanwhile, the use of synthetic chemicals as priming agents for seeds under stress conditions has major negative environmental concerns. Therefore, this work is conducted to overcome the negative effects of salinity on seed germination and growth of *C. lanatus* through seed priming in algal extracts. The study hypothesized that priming of *C. lanatus* in extracts of the seaweed *Ulva lactuca* could improve seed germination and plant growth under the impact of salinity, probably via increasing the content of photosynthetic pigments, antioxidants and other bioactive constituents.

## Materials and methods

### Plant materials

#### Seaweed collection

Fronds of *U. lactuca* L. were collected from the coastal area of the Red Sea coast, near Hurghada, Egypt, in January 2021, and washed with tap water to remove any salt residues or other impurities. Then, the samples were dried and ground into fine powder with a Wiley mill, and kept in paper bags at room temperature until used. *Citrullus lanatus* var. Colocynthoides seeds were kindly provided by the Agricultural Research Center (ARC), Giza, Egypt.

### Seaweed analysis

#### Assay of carbohydrates, lipids, proteins and glycine betaine

Soluble sugars were extracted from *U. lactuca* fronds according to the method adopted by Upmeyer and Koller^29^. To estimate insoluble sugars, the residue left after the extraction of soluble sugars was hydrolyzed by reflux in 0.2 N H_2_SO_4_ in a boiling water bath for 1 h^30^. Carbohydrate fractions were determined by the anthrone method^31^. The lipid content of seaweed was estimated by extracting an aliquot of 3 g of the powdered fronds in petroleum ether for 6 h in the Soxhlet system according to AOAC^40^, and the extraction was continued using as a solvent^32^. The soluble protein content of the alga was determined according to the method of Lowry et al.^33^, while total protein content was calculated by multiplying the Kjeldahl nitrogen content^34^ by 6.25. Assay of glycine betaine (GB) was carried out according to the method of Gorham^35^. Briefly, leaf extract was prepared by chopping 0.5 g of leaves in 5 mL of toluene-water mixture (0.05% toluene). The contents were shaken for 24 h at 25 °C. After filtration, 0.5 mL of the extract was mixed with 1 mL of 2 N HCl and 0.1 mL of potassium tri-iodide solution (containing 7.5 g iodine and 10 g potassium iodide in 100 mL of 1 N HCl) and the mixture was shaken in an ice-water bath for 90 min, and then 2 mL of ice-cooled water was added. After gentle shaking, 10 mL of dichloromethane (chilled at − 10 °C) was poured into the above mixture. By passing a continuous stream of air for 1–2 min, two layers were separated, the upper aqueous layer was discarded, and the absorbance of the organic layer was recorded at 365 nm. The concentration of GB was estimated by using a standard curve of GB in the range of 0–15 mg.

#### Proximate analysis of the seaweed

The detection of secondary metabolites (flavonoids, phenolics, alkaloids, saponins, tannins, and terpenoids) in *U. lactuca* aqueous extract was performed using the method of Sofowora^36^.

#### Quantitative determination of phenolic compounds

The phenolic content of *U. latuca* was determined using an Agilent-1100 HPLC system equipped with a quaternary gradient pump unit, an ultraviolet (UV) detector at 320 nm and a Zorbax Eclipse XDB-C18 analytical column (Agilent, USA) of 150 × 406 mm, 5 µm particle size. Elution was carried out at a flow rate of 0.075 mL min^−1^ at 23 °C. The mobile phase consisted of 8% acetonitrile, 22% isopropyl alcohol, and 70% formic acid solution (1%). All dissolved standards and samples were filtered through a 0.22 μm syringe filter before HPLC analysis. Seaweed aqueous extracts were frozen at − 20 °C for 24 h before drying in a frieze dryer, and the residues were dissolved in methanol (HPLC grade) before injecting into the HPLC; the injection volume was 20 μl. Identification of phenolic compounds was made by comparing the relative retention times of the sample peaks with those of the reference standards.

### Preparation of *U. lactuca* seaweed aqueous extract

One hundred grams of *U. lactuca* powder were extracted by soaking in 1000 mL distilled water for 24 h, with shaking using an orbital shaker at 25 °C; the mixture was then centrifuged at 5000 rpm for 20 min to remove the debris. The extract was filtered through Whatman No. 4 filter paper, and the final volume was completed to 1 L with distilled water to obtain the stock extract (10% w/v). The resulting extract was stored at − 4 °C until used.

### Effect of seaweed extracts on seed germination and plant growth of *C. lanatus*

The experiment was conducted during 2021/2022 at the Faculty of Science, Al-Azhar University (Girl Branch), Egypt. Plastic pots (20 cm in diameter and 20 cm in height) were filled with 3 kg of a clay/sand mixture (1:1) with the addition of the recommended doses of ammonium sulphate, ammonium nitrate and potassium sulphate fertilizers before sowing. The chemical analysis of the experimental soil mix was conducted at the beginning and the end of the experiment. Soil–water extracts were prepared by soaking 100 g of soil in 500 mL of distilled water for 1:5, soil extract and the filtrate was used for mineral analysis^37^. Soil chemical analysis revealed the following characteristics: pH (7.82–8.27), EC (0.36–0.56 dS m^−1^), and the mineral content (meq L^−1^): Ca^2+^ (12.455–13.23), Mg^2+^ (4.165–13.27), Na^+^ (26.63–36.44), K^+^ (2.32–2.62), CO_3_^2−^ (98.86–68.48), HCO_3_^−^ (779–752), S (2.21–2.42) and Cl^−^ (12.16–15.25) for the pre-and post-cultivation soil samples, respectively; the water holding capacity was up to 13.0%^38^.

Twenty seeds of *C. lanatus* were sown in each pot and planted in April after soaking for 6 h in *U. lactuca* extracts at four concentrations (0, 3, 5, and 8%), The pots were watered with four salinity levels (0,100, 200, and 300 mM NaCl) every 7 days until the end of the experiment. After complete emergence (10 days), number of seedlings was counted^39^, and seedlings were sequentially thinned to five per pot across the following five days. Pots were arranged in a randomized complete block design with three replicates in the greenhouse at 34 °C on average with 16 h of light and 8 h of darkness.

### Plant harvesting and analysis

#### Assay of plant growth and photosynthetic pigments

Three representative plant samples were taken from each treatment for measuring of growth traits after 45 days of growth: shoot length (cm), root length (cm), and the number of branches were measured; leaf area (cm2), was measured using leaf area meter (SYSTRONICS Leaf Area Meter-211). Photosynthetic pigments (chlorophyll a [Chl a], chlorophyll b [Chl b] and carotenoids [C(x + c)] were quantitatively determined spectrophotometrically according to the procedure adopted by Metzner et al.^40^ and, Abbas^41^. An aliquot of the fresh leaf tissue (0.5 g) was homogenized in a mortar with 10 mL of 80% acetone; the homogenate was centrifuged at 3000 rpm for 15 min at room temperature, and the supernatant was stored at 4 °C. The absorbance of the extract was measured at 645, 663 and 480 nm using a spectrophotometer (VEB Carl Zeiss). Chlorophyll a, chlorophyll b, and carotenoids were determined as µg g^−1^ leaf fresh weight using the following equations:

$$\begin{gathered} {\text{Chl a }}\left( {\upmu {\text{g mL}}^{{ - {1}}} } \right) = { 1}0.{\text{3 E}}_{{{663}}} - 0.{\text{918 E}}_{{{645}}} \hfill \\ {\text{Chl b }}\left( {\upmu {\text{g mL}}^{{ - {1}}} } \right) = { 19}.{\text{7 E}}_{{{645}}} - {3}.{\text{87 E}}_{{{663}}} \hfill \\ {\text{Carotenoids }}\left( {\upmu {\text{g mL}}^{{ - {1}}} } \right) = { 4}.{\text{2 E}}_{{{48}0}} - \left( {0.{\text{264 Chl a}} + 0.{\text{426 Chl b}}} \right) \hfill \\ \end{gathered}$$

The concentrations of chlorophylls and carotenoids were expressed as mg g^−1^ fresh weight (FW) of plant material.

#### Estimation of *C. lanatus* antioxidant enzyme activities

An aliquot of the fresh leaves (0.5 g) was homogenized in liquid nitrogen with the addition of 5 mL of 0.2 mol L^−1^ of sodium phosphate buffer solution (pH 7.8). Homogenates were centrifuged for 20 min at 4 °C, and supernatants were immediately used to determine enzyme activity. Total soluble protein content of the leaves was determined according to the procedure of Bradford and Williams^42^. Superoxide dismutase (SOD) activity was assayed in terms of the extent of inhibition of the photochemical reduction of p-nitro blue tetrazolium chloride (NBT)^43^. Catalase (CAT) activity was determined based on the rate of disappearance of H_2_O_2_, as measured by the decline in absorbance at 240 nm^44^. Peroxidase activity (POD) was calculated from the rate of formation of guaiacol dehydrogenation product and was expressed as mmol GDHP min^−1^ mg^−1^ protein^45^.

### Data analysis

The data were subjected to two-way ANOVA according to Snedecor and Cochran^46^ using SPSS ver. 26.0, and considering p < 0.05 as a significant level. Tukey's HSD is used to test differences among sample means for significance. Spearman correlation was done by Sigmaplot 12.5, to assess the effects of seaweed and salinity on germination, seedling growth and plant performance.

### Ethical approval

All the steps of experimentation on *C. lanatus* var. Colocynthoide plants, including the collection of plant material, are in compliance with relevant Institutional, National, and International guidelines. The greenhouse studies were conducted in accordance with local legislation and with permissions from our institutes and complied with the IUCN Policy Statement.

## Results and discussions

### Chemical composition of *Ulva lactuca*

Results of the biochemical analyses of *U. lactuca* fronds indicate that the algal biomass contained (one dry weight basis): 0.93 mg g^−1^ total carbohydrates, 3.01 mg g^−1^ of lipids, and 3.21 mg g^−1^ protein, in addition to appreciable content of glycine betaine (5.13 mg g^−1^) and total phenolics (0.0188 mg g^−1^). This result is in agreement with Latique et al.^47^ who found that *Ulva rigida* extract is rich in soluble sugars, polyphenols, and proteins which might be necessary for the stimulation of antioxidant enzymes. Glycine betaine is an osmoregulatory molecule that enables plant cells to cope with salt stress^48^.

The aqueous extract of *U. lactuca* contained appreciable content of saponins, alkaloids, and tannins along with high content of flavonoids and phenolics but lacks terpenes. Hence, the high phenolic and flavonoid content of *U. lactuca* extract may account for its high efficiency in the enhancement of *C. lanatus* and alleviation of the impact of salt stress.

Nine free compounds were identified in the aqueous extract of *U. lactuca* extract (Table 1) by reverse-phase high-performance liquid chromatography (HPLC). These compounds were identified based on their relative retention times. Ascorbic acid and coumaric acid amounted to about 48.59 and 36.23 µg g^−1^ DW, respectively, and were the two main components in *U. lactuca* extract. Caffeic, ferulic, protocatechuic and pyrogallic acids, and resorcinol all recorded moderate amounts of 29.3, 19.67, 25.92, 19.72, and 25.3 µg g^−1^ DW, respectively. But, chlorogenic and salicylic acids were detected with relatively low quantities of 15.33 and 16.97% μg g^−1^ DW, respectively. *U. lactuca* methanolic extract has been reported to contain phenolics, flavonoids, alkaloids, and tannins^49^. Phenolics are identified as the largest group that can cause a stabilized electrolytic effect, as the substances are soluble in water^50^. Phytohormones, amino acids, polyphenols, carbohydrates, alginates, essential macro- and micronutrients, betaines, and vitamins are among the physiologically active compounds found in seaweed extracts that stimulate plant growth and development^28^. Klejdus et al.^51^ and Kovacik et al.^52^ who found a variety of phenolic compounds in algae. Algae are rich in biologically active components such as phenolic compounds, polysaccharides, hormone-like substances, and proteins^53^.

**Table 1:** Fractionation of phenolic content of *U. lactuca* aqueous extract using HHPLC (each values is the means of three replicates ± SE.).

Standard phenolic compounds	Retention time(min)	Concentration (μg g^−1^ dry weight)
Pyrogallic acid	9.697	19.72 ± 1.04
Resorcinol	13.623	25.30 ± 1.45
Protocatechuic acid	14.217	25.92 ± 1.25
Chlorogenic acid	15.831	15.33 ± 0.78
Caffeic acid	19.520	29.3 ± 1.32
Coumaric acid	21.235	36.23 ± 2.02
Ferulic acid	24.321	19.67 ± 1.11
Salicylic acid	31.129	16.97 ± 0.43
Total concentration		188.64
Ascorbic acid	22.532	48.59 ± 1.48

### Effect of *U. lactuca *an aqueous extract on *C. lanatus* seed germination

**Figure 1:**
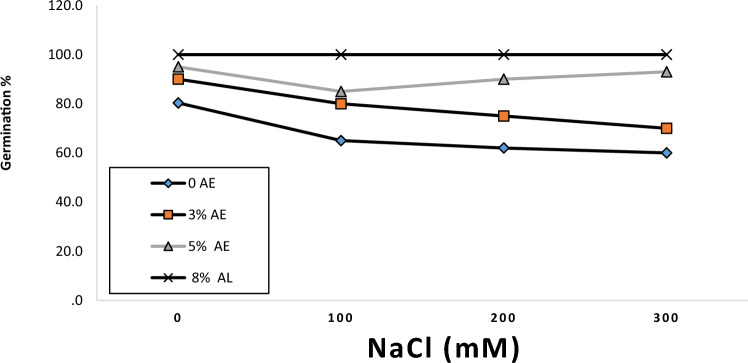
Effect of aqueous extract of *U. lactuca* (UL) on seed germination of *C. lanatus*. Each column value is the mean of three/five replications ± SE. Means with the same letter are non-significantly different (p < 0.05.

Figure 1 shows that priming of *C. lanatus* seeds in *U. lactuca* extracts significantly improved germination of seeds, and the increase was most evident at high salinity, Increasing concentration of algal extract during priming from 0 to 0.8% increased. Germination percentage of *C. lanatus* seeds by 24.5%, 53.9%, 61.3%and 66.7% at 0.0, 100, 200 and 300 mM NaCl salinity, respectively. Increasing NaCl salinity reduced seed germination and the magnitude of reduction differed according to the concentration of the priming algal extract. Whereas increasing salinity from 0 to 300 mM NaCl led to progressive 20.3% reduction in seed germination in absence of algal extract, the reductions were relatively mild and amounted to 80.3%, 65.0%, 60.0 and 60.0% at 3%, 5% and 8% algal extract, respectively. The germination in *C. lanatus* was affected significantly by algal concentrations (F = 44.73, *p* < 0.000) and salt concentrations (F = 14.07, *p* < 0.000), while, there was interaction within algal concentrations and salt concentrations (F = 6.25, *p* < 0.02) (Table 2). Salt stress presents a threat to *C. lanatus* germination, growth parameters, and photo/biosynthetic materials. Therefore, Algal extracts from *U. lactuca* can be used as priming practices to promote *C. lanatus* cultivation under salinity stress. Seeds are usually soaked in an osmotic solution that allows them to imbibe water and go through the first stages of germination^54^. Seed pretreatment with algal extract that contains bioactive substances can stimulate the metabolic process in *C. lanatus* seeds under the impact of salinity stress. Seed presoaking can stimulated the antioxidant enzyme activities that play a vital role in *C. lanatus* resistance against salt stress. The beneficial et of algal extract in improvement of seed germination percentage and the subsequent plant growth of *C. lanatus* under the impact of salinity can be attributed to its effect on seed surface permeability to salts, and its content of bioactive components in *U. lactuca* extract of glycine betaine and phenolics and ascorbic acid could potentially participate in the alleviation of salinity stress. Thus, priming of *C. lanatus* seeds with *U. lactuca* extract could induce many physiological and biochemical changes that can stimulate seeds to germinate faster and increase the germination percentage of *C. lanatus*. Algal extracts increase seed germination and seedling growth in several plants spices with alleviation of the impact of a biotic stress^55–58^ and fruit production^59^.

**Table 2:** Two-way ANOVA showing the effect of the main factors: algal extract (AE) and salinity (Saln.) and their interaction on seed germination and plant growth and performance of *C. lanatus var.* Colocynthoides.

Variable and source of variation	df	F	p	Variable and source of variation	df	F	p
Germination %				Root length			
AE	3	44.73	0.00	AE	3	3.40	0.02
Saln	3	14.07	0.00	Saln	3	18.01	0.00
AE × Saln	9	0.846	0.54	AE × Saln	9	0.304	0.930
Shoot length				Number of branches			
AE	3	27.27	0.00	AE	3	6.05	0.002
Saln	3	16.05	0.00	Saln	3	5.74	0.00
AE × Saln	9	0.563	0.756	AE × Saln	9	0.759	0.60
Leaves area							
AE	3	17.54	0.00				
Saln	3	11.63	0.009				
AE × Saln	9	0.96	0.46				

### Effect of *U. lactuca *extract on the growth of *C. lanatus*

**Figure 2:**
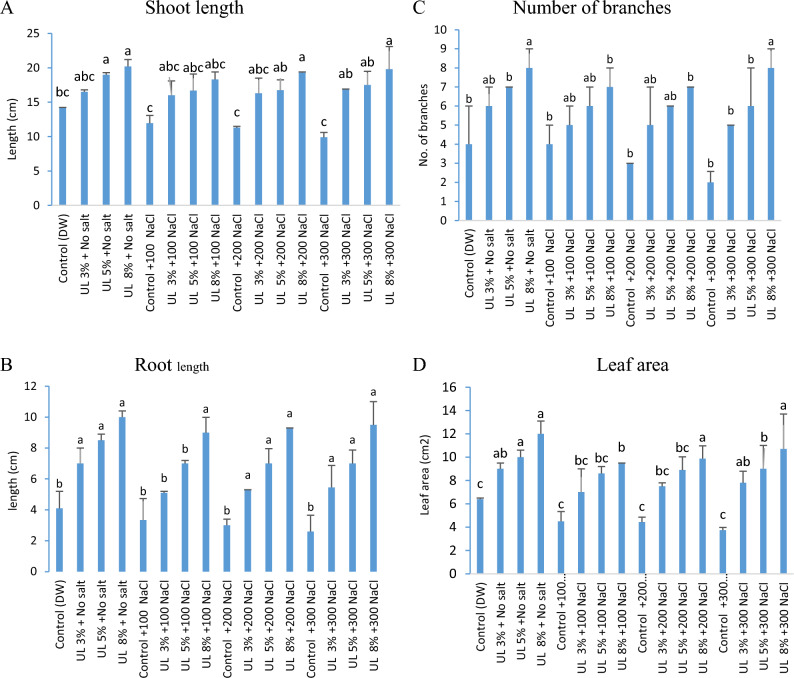
Effect of aqueous extract of *U. lactuca* (UL) on growth of *C. lanatus.* (**A**) Shoot length, (**B**) root length, (**C**) number of branches, (**D**) leaf area. Each column value is the mean of three/five replications ± SE. Means with the same letter are non-significantly different (p < 0.05).

Shoot length, root length, number of branches and leaf area of *C. lanatus* significantly increased with priming in *U. lactuca* extract under various levels of sodium chloride as compared to its controls (no salt-no extract). Figure 2 shows that priming of *C. lanatus* seeds in *U. lactuca* extracts at 0.8% significantly improved *C. lanatus* by 42.25%, 53.14%, 70.80% and 100.0% (shoot length), 143.90%, 169.16%, 210.0% and 265.38% (root length), 100.0%, 75.0%, 133.33% and 300.0% (number of branches), 87.50%, 111.11%, 22.8% and 140.0% (leaf area) at 0.0, 100, 200 at 0.0, 100, 200 and 300 mM NaCl salinity, respectively. Whereas increasing salinity led to progressive 30.28%, 36.58%, 50.0% and 41.56% reduction in shoot length, root length, number of branches and leaf area from 0 to 300 mM NaCl in absence of algal extract. The reductions were relatively mild and amounted to 15.85%, 20.42%, and 30.2% (shoot length), 18.53%, 26.82%, and 36.58% (root length), 0.0%, 25.0%, and 50.0% (number of branches), 29.68%, 30.78% and 41.56% (leaf area) at 3%, 5% and 8% algal extract, respectively. The traits in *C. lanatus* of shoot length were affected by algal concentrations significantly (F = 27.27, *p* < 0.000) and salt concentrations (F = 16.05, *p* < 0.000), root length by salt concentrations (F = 18.015, *p* < 0.000), number of branches by algal concentrations (F = 6.06.575, *p* < 0.02) and by salt concentrations (F = 5.7, *p* < 0.00) and leaf areas by algal concentrations (F = 17.54, *p* < 0.0000) and salt concentrations (F = 11.64, *p* < 0.0000) (Fig. 2, Table 2). The highest increment in shoot length was obtained from *U. lactuca* extract at 8% concentration to reach about 42.25, 53.14, 70.80 and 100.0%, of 0, 100, 200, and 300 mM NaCl, respectively. The greatest increment was achieved at 8% of root length reaching about 143.9, 169.16, 210.0 and 265.38%, of 0, 100, 200, and 300 mM NaCl, respectively. The use of *U. lactuca* extract at 8% increased the total number of branches per plant significantly to give about 100.0, 75.0, 133.3 and 300.0%, of 0, 100, 200, and 300 mM NaCl, respectively. The leaf area increased significantly with the application of *U. lactuca* extract, and the highest increment was attained at 8% algal extract to give about 87.5, 111.1, 122.8 and 186.1%, with all NaCl levels of 0,100, 200, and 300 mM, respectively.

The priming of *C. lanatus* seeds in *U. lactuca* aqueous extract improved plant growth and photosynthetic pigments significantly under the impact of salinity. Shoot length, root length and leaf area as well as the content of photosynthetic pigments have been reported to increase in the salinized plants in response to presoaking of seeds in *Ulva fasciata* extract^60,61^. The beneficial effect of primed cherry tomato seeds in *Ulva flexuosa* extract on could be attributed to the high concentration of glycine betaine in the algal extract^62^. In support to this claim, exogenous application of glycine betaine significantly increased plant height and the number of leaves of *Dalbergia odorifera* seedlings under mild and severe salinity^63^.

### Effect of *U. lactuca* extracts on photosynthetic pigments of *C. lanatus*

**Figure 3:**
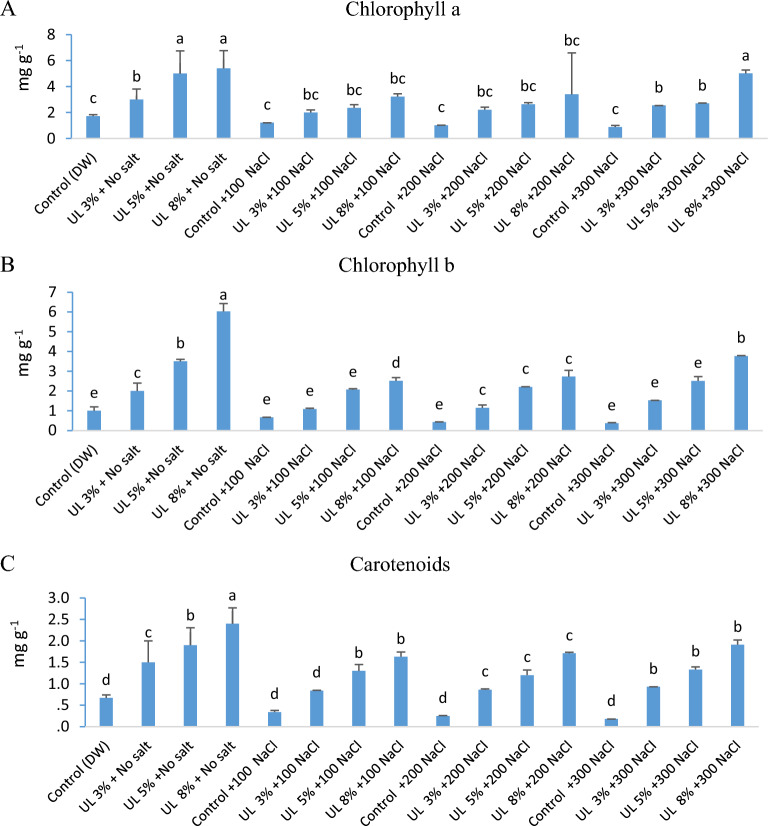
(**a**–**c**) Photosynthetic pigments (mg g^−1^ FW) of *C. lanatus* (UL) affected by different algal extract concentrations. Means with the same letter are not significantly different from each other (p 0.05 ANOVA).

The effects of salinity and algal priming on leaf photosynthetic pigments of *C. lanatus* were substantial (Table 3), with relatively stronger effect of salinity than algal priming and very highly significant interaction, except for the non-significant interaction on carotenoid content. The content of chlorophyll a, chlorophyll b, and carotenoids increased progressively by increasing the concentration of *U. lactuca* extract. The increase in chlorophyll a, chlorophyll b, and carotenoids at 8% aqueous algal extract amounted to 469%, 918%, and 961% respectively above the non-treated control (Fig. 3). Our results are in agreement with Ahmed et al.^64^ who found that the content of chlorophylls a and b and carotenoids in cotton leaves was significantly improved by algal extract as compared to the control. The application of *U. rigida* extract to wheat plants under salt stress conditions led to a significant increase in Total Chl (T-Chl), Chl-a, and Chl-b^65,78^. Leaf pigments are a major physiological attribute that is directly related to the photosynthesis process under various environmental conditions^66^. This increase in chlorophyll content could be a result of the protection of chlorophyll from degradation that might be attributable to betaines of the seaweed extract^67^. Seaweed extracts by virtue of their high mineral content, particularly, magnesium and can increase leaf chlorophyll and carotenoid concentration^68^. Application of extracts of *Chlorella liposies* and *Spirolina maxima* to wheat plants under salt-stress caused a significant increase in the content of Chl-a and Chl-b^69–71^. The application of *Ulva fasciata* extracts significantly increased the production of *Vigna sinensis* and *Zea mays* growth and photosynthetic pigments under salt stress conditions^72^.

**Table 3:** Two-way ANOVA showing the effect of the main factors: algal extract (AE) and salinity (Saln.) and their interaction on pigments and Enzyme performance of *C. lanatus var.* Colocynthoides.

Variable and source of variation	df	F	p	Variable and source of variation	df	F	p
Pigments				Enzymes			
Chlorophyll a				SOD			
AE	3	38.95	0.00	AE	3	6.22	0.002
Saln	3	53.50	0.00	Saln	3	11.51	0.00
AE × Saln	9	5.45	0.01	AE × Saln	9	5.837	0.00
Chlorophyll a				CAT			
AE	3	223.89	0.00	AE	3	66.99	0.00
Saln	3	361.87	0.00	Saln	3	61.30	0.00
AE × Saln	9	32.67	0.00	AE × Saln	9	22.38	0.00
Carotenoids				POD			
AE	3	68.20	0.00	AE	3	185.04	0.00
Saln	3	106.28	0.00	Saln	3	46.63	0.00
AE × Saln	9	0.766	0.602	AE × Saln	9	36.29	0.00

### Effect of *U. lactuca* extracts on superoxide dismutase (SOD), catalase (CAT), and peroxidase (POD) activities of *C. lanatus*

**Figure 4:**
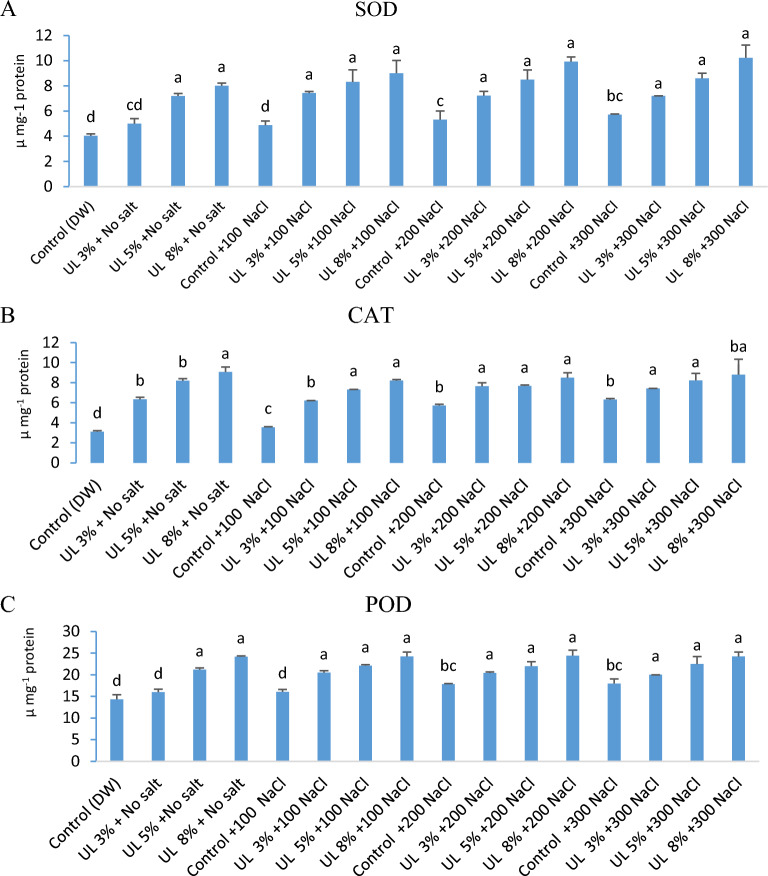
(**a**–**c**) Superoxide dismutase (SOD), catalase (CAT), and peroxidase (POD) (µ mg^−1^ protein) of *C. lanatus* affected by different algal extract concentrations (UL). Means with the same letter are not significantly different from each other (p 0.05 ANOVA).

The results in Fig. 4a–c revealed that the algal extract at 3%, 5%, and 8% significantly induced SOD, CAT, and POD activities under the tested salt levels. The maximum activity of SOD, CAT, and POD was recorded at 8% algal extract with 10.23, 8.08, and 22.5 µg^−1^ protein, respectively. The increasing values at 8% algal extract were 98.51, 84.63, 86.59 and 78.85% (SOD), 191.64, 131.01, 58.43, and 39.39% (CAT) and 68.62, 51.0, 36.94 and 34.71% (POD), in NaCl levels of 0,100, 200, and 300 mM respectively compared with it’s the control. The SOD activity of *C. lanatus* was affected significantly by algal concentrations (F = 6.22, *p* < 0.02) and salt concentrations (F = 11.51, *p* < 0.000), while, there was an interaction between algal concentrations and salt concentrations (F = 5.83, *p* < 0.000). The CAT was affected significantly by algal concentrations (F = 66.99, *p* < 0.00) and salt concentrations (F = 61.30, *p* < 0.000), and there was a significant interaction between algal concentrations and salt concentrations (F = 22.28, *p* < 0.000). The POX was affected significantly by algal concentrations (F = 185.04, *p* < 0.00) and salt concentrations (F = 64.63, *p* < 0.000), and there was a significant interaction between algal and salt concentrations (F = 36.29, *p* < 0.000) (Table 3). The algal extract could enhance the activity of SOD, CAT, and POD in *C. lanatus* along with improving salt tolerance that boosts the plant development under stress conditions. Salinity tolerance is strongly associated with enhanced antioxidant enzyme activities as well as high content of non-enzymatic antioxidants^73^. The role of antioxidant enzymes in salt-resistance has been reported in many plant species (Arabidopsis, barley, pea, and tobacco)^74–76^. Macroalgae have been recognized as a rich source of antioxidants^77^. Algal extract of *U. rigida* antagonizes the harmful effects of abiotic stress by increasing the activity of antioxidant enzymes^78^. *Ulva rigida* extract led to a significant promotion in antioxidant enzyme activity in salt-stressed wheat plants^79^. Superoxide dismutase (SOD) is the first enzyme that acts against reactive oxygen species^80^. A significant enhancement in catalase (CAT) and superoxide dismutase (SOD) activities was observed in wheat plants treated with 25% of *U. rigida* extract under salt stress conditions^81^. Superoxide dismutase, catalase and peroxidases as well as ascorbate peroxidase have been claimed to protect plants against the harmful effects of reactive oxygen species^82,83^. The content of soluble sugars, polyphenols, and proteins in *Fucus spiralis* extract may account for the increased salt resistance of durum wheat, probably via stimulation of antioxidant enzymes^47^.

Furthermore, germination and growth traits were positively correlated to enzyme activity and pigments (Table 4). Shoot length, root length, and germination were associated and positively correlated with chlorophyll (b) by 0.984, 0.984, and 0.943, respectively. The number of branches and leaf area were correlated positively with carotenoids by 0.985, and 0.996, respectively. Shoot length, root length, Number of branches and leaf area recorded the strongest correlation with CAT enzyme by 0.924, 0.895, 0.881, and 0.887, respectively. Germination recorded a positive correlation with SOD, CAT, POD enzymes by 0.697, 0.826, and 0.780, respectively. Seed priming boosts pre-germination metabolic activities, boosts antioxidant system activity, and speeds up membrane mending^84^. The promoting priming effects of algal extracts in plant growth can contributed to the supply of plant nutrients from the soils^85^. Some of these elements are directly related to leaf pigments or catalyze some physiological process that leads to promoting the production of biochemical substitutes^86^. Seaweed extracts enhanced the biochemical constituents in crops^87^. Algal extracts can enforce different physiological and biochemical mechanisms to improve the salinity tolerance of plants^60^. Seaweed extract of *U. rigida* can increase salt stress tolerance and protect wheat plants against oxidative deterioration^88^. Macroalgae could enhance plant salt tolerance through the increasing antioxidant compounds, compounds such as ascorbic acid and polyphenols, which might be necessary for the stimulation of antioxidant enzymes^47^. Algal extracts can relieve the effects of salt stress through the activation of metabolic pathways that contribute to promoting plant growth and yield^89^. The enhancement of plant salt resistance was associated with an increase in water use efficiency, photosynthetic activity, phenolic compounds, and ROS scavenging activity. Glycine betaine is involved in mitigating salt stress in plants through osmotic adjustment and ROS scavenging^90^. *Ulva rigida* extract is rich in glycine betaine, which delays the loss of photosynthetic activity by inhibiting chlorophyll degradation^67^. Exogenous application of glycine betaine significantly increased plant height and number of leaves of *Dalbergia odorifera* under mild and severe salinity stress conditions^63^. It could be concluded that the aqueous extract of the seaweed *U. lactuca* can boost seed germination percentage, plant growth, photosynthetic pigments and performance of *C. lanatus* under salt stress conditions by virtue of their contents of bioactive components and biochemical parameters, they could stimulate the tolerance response of *C. lanatus* toward biotic stress of salts. Therefore, this research advised breeders to use seaweed extract as an effective seed priming technique to promote *C. lanatus* germination and growth is an effective under salt stress conditions for increasing plant productivity.

**Table 4:** Correlation among growth traits, pigments and Enzyme performance of *C. lanatus var.* Colocynthoides under salinity condition and application of *U. lactuca* aqueous extract.

	Shoot length	Root length	No. of branches	Leaf area	Germination	SOD	CAT	POD	Chlorophyll a	Chlorophyll b	Carotenoid
Shoot length		0.967	0.954	0.965	0.906	0.724	0.924	0.809	0.965	0.979	0.956
Root length			0.993	0.984	0.956	0.722	0.895	0.804	0.976	0.984	0.984
No. of branches				0.986	0.956	0.716	0.881	0.800	0.975	0.985	0.985
Leaf area					0.946	0.648	0.887	0.739	0.996	0.987	0.996
Germination						0.697	0.826	0.780	0.931	0.943	0.939
SOD							0.818	0.950	0.621	0.712	0.629
CAT								0.874	0.879	0.912	0.868
POD									0.709	0.803	0.726
Chlorophyll a										0.976	0.991
Chlorophyll b											0.979
Carotenoid											

## Data Availability

The datasets used during the current study are available from the corresponding author upon reasonable request.
